# Use of handheld computers in clinical practice: a systematic review

**DOI:** 10.1186/1472-6947-14-56

**Published:** 2014-07-06

**Authors:** Sharon Mickan, Helen Atherton, Nia Wyn Roberts, Carl Heneghan, Julie K Tilson

**Affiliations:** 1 Nuffield Department of Primary Care Health Sciences, University of Oxford, Oxford, UK; 2 Division of Biokinesiology and Physical Therapy, University of Southern California, Los Angeles, USA

**Keywords:** Handheld computers, Smartphone, Information-seeking behaviour, Evidence-based practice, Knowledge translation, Clinical decision support systems, Clinical guidelines, Diagnostic decision making

## Abstract

**Background:**

Many healthcare professionals use smartphones and tablets to inform patient care. Contemporary research suggests that handheld computers may support aspects of clinical diagnosis and management. This systematic review was designed to synthesise high quality evidence to answer the question; Does healthcare professionals’ use of handheld computers improve their access to information and support clinical decision making at the point of care?

**Methods:**

A detailed search was conducted using Cochrane, MEDLINE, EMBASE, PsycINFO, Science and Social Science Citation Indices since 2001. Interventions promoting healthcare professionals seeking information or making clinical decisions using handheld computers were included. Classroom learning and the use of laptop computers were excluded. Two authors independently selected studies, assessed quality using the Cochrane Risk of Bias tool and extracted data. High levels of data heterogeneity negated statistical synthesis. Instead, evidence for effectiveness was summarised narratively, according to each study’s aim for assessing the impact of handheld computer use.

**Results:**

We included seven randomised trials investigating medical or nursing staffs’ use of Personal Digital Assistants. Effectiveness was demonstrated across three distinct functions that emerged from the data: accessing information for clinical knowledge, adherence to guidelines and diagnostic decision making. When healthcare professionals used handheld computers to access clinical information, their knowledge improved significantly more than peers who used paper resources. When clinical guideline recommendations were presented on handheld computers, clinicians made significantly safer prescribing decisions and adhered more closely to recommendations than peers using paper resources. Finally, healthcare professionals made significantly more appropriate diagnostic decisions using clinical decision making tools on handheld computers compared to colleagues who did not have access to these tools. For these clinical decisions, the numbers need to test/screen were all less than 11.

**Conclusion:**

Healthcare professionals’ use of handheld computers may improve their information seeking, adherence to guidelines and clinical decision making. Handheld computers can provide real time access to and analysis of clinical information. The integration of clinical decision support systems within handheld computers offers clinicians the highest level of synthesised evidence at the point of care. Future research is needed to replicate these early results and to identify beneficial clinical outcomes.

## Background

Increasing numbers of healthcare professionals use handheld computers that offer instant access to vast amounts of information via the internet and healthcare applications (apps) [1]. Over the last 10 years there has been a rapid and accelerating rate of innovation in handheld computers, from personal digital assistants (PDAs) towards more powerful, versatile and internet connected devices. As the rate of adoption of handheld computers has increased, individual patterns of usage have moved from that of communication and personal diary management towards information seeking and decision support [2]. Today’s clinicians can use handheld computers to search the internet for evidence and guidance on drugs and clinical conditions, use clinical decision support systems (CDSS) and access highly detailed patient information from clinical and laboratory investigations.

At the same time, there has been a change in the acceptance of using handheld computers in healthcare settings. Now, most students and many professionals are enthusiastic about using smartphones and tablet computers, and they take them wherever they go [3]. Along with this increasing adoption of handheld computers, there has been a massive growth in the volume of synthesized research information, healthcare oriented apps, databases and CDSSs.

This has also sparked an increased production of feasibility research, which has yet to recommend strategies for engagement, efficacy or effectiveness of mobile health initiatives [4]. While both early and current systematic reviews offer tentative and sceptical conclusions, there is equipoise in the literature. A systematic review of the use of PDAs in clinical decision making reported an increase in data collection quality and concluded that the use of decision support software improved the appropriateness of diagnostic and treatment decisions [2]. In a broader and contemporary systematic review of mHealth technologies, modest benefits were reported for improved clinical diagnosis and management support, and mixed outcomes were reported for efficient and accurate documentation [3]. Further, there was no clear benefit for educational interventions and some evidence of reduced quality of clinical assessment, when using mobile technology based photos.

When healthcare professionals communicate with patients, there is high quality evidence to support the use of mobile phones to transmit short message service (SMS) reminders to improve attendance at health care appointments [5,6]. Further, text messaging interventions were shown to increase adherence to antiretroviral therapy in low-income settings and increased smoking cessation in high income settings [7].

An early review of computerised, rather than mobile, CDSSs for prescribing, described effectiveness in initiating and monitoring therapy, but provided little evidence on their impact in specific clinical settings [8]. A later review reported improved processes of care in 60% of included studies but improved patient outcomes in only 20% of studies [9]. It is not clear whether incorporating these computerised systems into mobile devices would produce similar results.

A literature and commercial review of mobile CDSSs reported medical professionals using a growing number of apps across a wide range of fields [10]. A systematic review of smartphone healthcare apps identified seven functional categories in which apps have been developed for use by healthcare professionals: diagnosis, drug reference, medical calculators, literature search, clinical communication, access to hospital information systems, and medical training [1]. A scoping review from a further five systematic reviews concluded that there is evidence for effective use of handheld computers by healthcare professionals across four key functions: providing easy and timely access to information, enabling accurate and complete documentation, providing instant access to evidence-based decision support and patient management systems, and promoting efficient work practices [11].

Most published studies to date describe the design, development and implementation of handheld computers using observational study designs [4]. In order to determine the benefits of integrating handheld computer use in healthcare practice, it is important to summarise and quantify results from the highest quality randomised controlled trials (RCTs) of effectiveness studies. Based on the functions identified in the earlier scoping review [11], it is timely to better understand whether healthcare professionals’ use of handheld computers facilitates information seeking and improved clinical decision making. The purpose of this review is to answer the research question “Does healthcare professionals’ use of handheld computers improve their access to information and support clinical decision making at the point of care?”.

## Methods

The protocol for this systematic review was registered with PROSPERO (CRD42011001632), updated and adhered to. http://www.crd.york.ac.uk/PROSPERO/display_record.asp?ID=CRD42011001632#.U7-vibFnDhA.

### Search strategy

We searched the following databases from 2001 to 19th August 2013: Cochrane Central Register of Controlled Trials (CENTRAL), MEDLINE, EMBASE, PsycINFO, Science Citation Index and Social Science Citation Index. The MEDLINE search strategy can be found in the Additional file 1. Reference lists of included studies were hand searched.

### Inclusion and exclusion criteria

We included studies whose participants were healthcare professionals using handheld devices in clinical settings. Interventions of interest were those investigating the use of handheld computers to promote healthcare professionals’ information seeking (outside of formal education courses), or to support informed clinical decision making. Our comparator was usual clinical practice. We excluded the use of laptops.

Study designs included were RCTs. The review was restricted to the English language. We searched from 2001 onwards to account for the changing nature of technology. We excluded studies that were presented as abstracts only, and where author contact confirmed the study had not been published in full.

### Study selection

Two authors (SM and JT) screened titles and abstracts. Full text articles were obtained for those selected and screened for inclusion (SM and HA). Where necessary, authors of studies were contacted for clarification of inclusion status.

### Data extraction

A data extraction form was designed and piloted by two authors (SM and HA) to record study design, country, device used, aim, participants, setting, intervention, comparator, primary and secondary outcome data (as reported by the systematic review authors). The same authors independently extracted data. Disagreements were resolved by discussion.

### Assessment of quality

Assessment of risk of bias was conducted at the study level using the Cochrane Risk of Bias tool [12]. Assessment was conducted independently by two authors (SM and HA) with disagreements resolved by discussion. Information on risk of bias status was used to aid interpretation of the included studies.

### Data synthesis

High levels of data heterogeneity and mixed data quality meant that statistical synthesis was not possible. We adopted a narrative approach to summarise the evidence for effectiveness according to the purpose for using the handheld computer.

## Results

**Figure 1:**
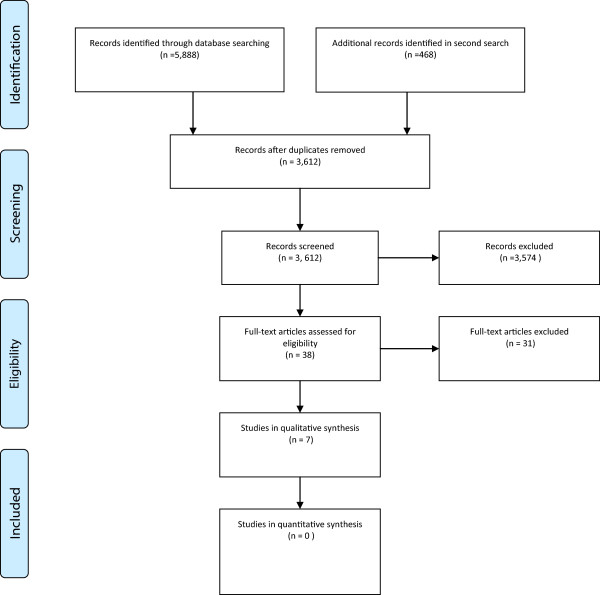
Flow diagram.

The combined search strategies identified 5,888 titles. After duplicates were removed, 3,612 titles were screened for eligibility. Thirty-eight full text articles were read, of which 31 did not meet the inclusion and exclusion criteria and therefore seven studies were retained for data extraction (see Figure 1).

Characteristics of the seven included studies are summarised in Table 1. All were RCTs, mostly designed as pilot studies with comparatively small numbers of participants (range 12-76 participants). Although we intended to include studies investigating smartphones and tablets, to represent the most current forms of handheld computers, all included studies investigated the use of PDAs. Three studies were conducted in USA, two in Canada and one each in France and Australia. In five studies, the intervention group used a PDA while the control group used paper-based resources. In two studies, both groups used a PDA, but the intervention group had access to a specific clinical decision support system (CDSS) or information tool that the control group did not. Healthcare participants were either medical (residents, fellows, and family, general and emergency physicians) or nursing professionals. Where students were included, they were using a PDA in a clinical environment.

**Table 1:** Characteristics of included studies

**Study, country**	**Participants, setting**	**Intervention**	**Comparator**	**Primary outcome**	**Secondary outcome**
Berner 2006 USA [13]	59 Internal medicine residents, University outpatient clinic	PDA with rule for gastrointestinal risk assessment when prescribing NSAIDS	PDA without rule for gastrointestinal risk assessment when prescribing NSAIDS	Difference in unsafe NSAID prescriptions	Identification of key risk factors for standardised patient case
Bochicchio 2006 USA [14]	12 1^st^ year critical care fellows, University hospital	PDA with John Hopkins Antibiotic Guide	No PDA, instructed to use written reference guides	Difference in mean score for knowledge test	Antibiotic decision accuracy
Farrell 2008 Australia [15]	76 nursing students, Medical-surgical wards	PDA with pharmacological information and training session	No training or PDA	Difference in mean score for pharmacology test	N/A
Greiver 2005 Canada [18]	18 Family physicians, Family practice (65 patients)	PDA with angina diagnosis software	Conventional care	Appropriate referral for cardiac stress testing at presentation, and nuclear cardiology after cardiac stress testing	Referral to cardiologists
Lee 2009 USA [19]	29 registered nurses, Hospital and ambulatory care (1874 patients)	PDA with CDSS for obesity diagnosis	PDA without CDSS for obesity diagnosis	Appropriate obesity related diagnosis	Missed obesity related diagnosis
Price 2005 Canada [16]	8 General practitioners, General practice (79 patients)	PDA with reminder for 5 preventive measures	Software provided after the study	Adherence to five guidelines	N/A
Roy 2009 France [17]	24 Emergency physicians, 10 emergency departments (1645 patients)	PDA with CDSS for pulmonary embolism	PDA used for data collection only; Paper based guideline material	Appropriate diagnostic strategy for pulmonary embolism	Adherence to recommended diagnostic testing Number of tests per patient

**Figure 2:**
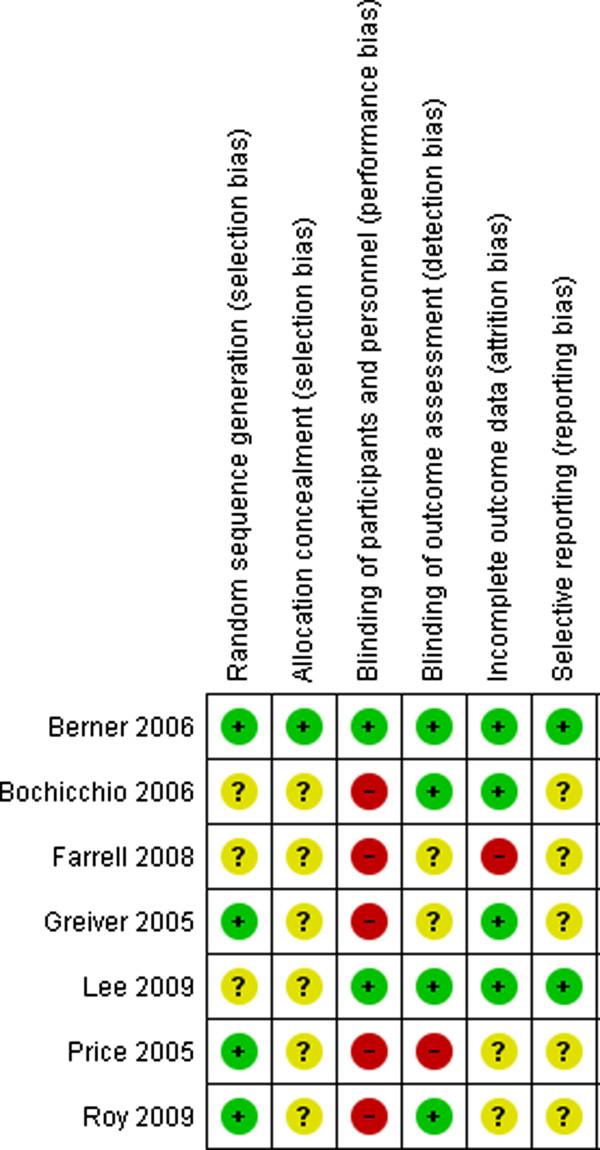
Risk of bias in included studies.

**Figure 3:**
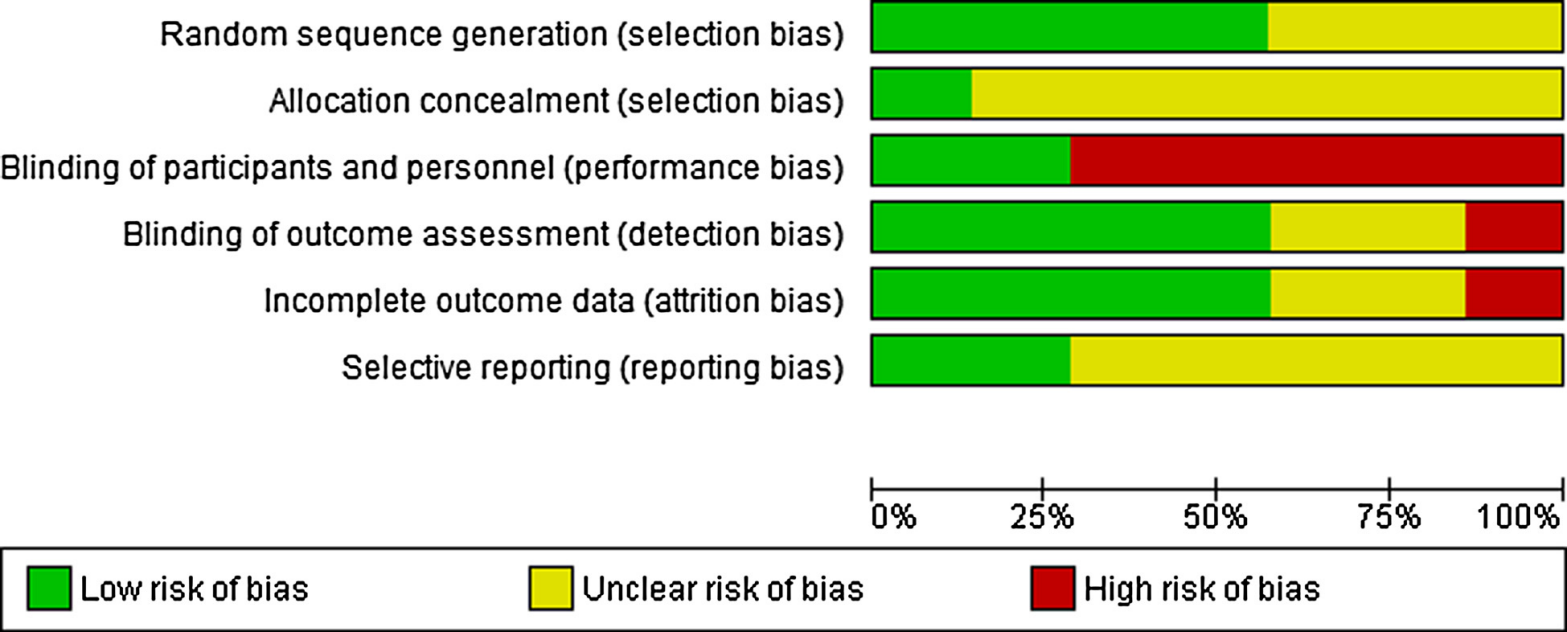
Risk of bias across individual domains of risk of bias.

The risk of bias assessment for included studies is shown in Figure 2 and summarised in Figure 3. The studies were largely at low or unclear risk of bias. Only one study [13] was at low risk of bias for all domains. The highest risk of bias occurred for blinding of participants, which is not always practical when investigating the use of an obvious handheld computer. Participants could not be blinded in 5 studies where the intervention group used a PDA and the control group did not. Blinding was achieved, however, in two studies where both intervention and control groups were using a PDA. Four studies ensured that the outcome assessment was blinded.

Heterogeneity of outcomes (Table 1) negated any quantitative synthesis or meta-analysis in this review. Instead, narrative summary was used to describe evidence of effectiveness of three distinct functions of handheld computers that emerged from the data: accessing information for clinical knowledge, adherence to safety and quality guidelines, and diagnostic decision making.

### Information for clinical knowledge

There is evidence from two pilot studies, one showing statistical significance [14], that when doctors and nurses used a handheld computer to access information in clinical environments, their clinical knowledge improved more than their peers who used traditional paper resources [14,15].

Medical Fellows allocated to use a PDA in hospital intensive care units increased their mean knowledge of infectious disease management more than those who used paper resources at both 3 months (p < 0.05) and 6 months (p < 0.01) [14]. PDA use also increased fellows’ antibiotic selection accuracy, a secondary aim of this project. Of the 125 antibiotic selections evaluated, fellows’ antibiotic selection accuracy improved from 66% (33/50) in the first 3 months, to 87% (65/75) in the second 3 month period. In the second study, pharmacological knowledge of 76 nursing students was tested before and after a 3 week clinical placement on rather than in medical and surgical wards. All demonstrated increased pharmacological knowledge, with those who used a PDA increasing their mean score twice as much as those who used only paper resources. However, this was not significant (p = 0.17) [15].

### Adherence to guidelines

Two feasibility RCTs examined the impact of healthcare professionals’ use of PDAs on adherence to safety and quality guidelines in clinical practice [13,16]. One identified a statistically significant result [13] and both found that the PDA promoted adherence to guidelines.

In one study, 59 internal medicine residents received a PDA [13]. The intervention group’s PDA included software supporting the use of a prediction rule for assessing gastro-intestinal risk when prescribing non-steroidal anti-inflammatory drugs (NSAIDs). Thirteen standardised patients (all with moderate risks for an adverse gastro-intestinal event) were included in regular clinics and their medical records and prescriptions were independently reviewed by clinicians blinded to the residents’ group assignment. Residents using a PDA with the prediction rule made fewer unsafe NSAID prescriptions (23% vs 45%, p < 0.05) and the mean proportion of cases per physician with unsafe prescriptions was significantly lower (0 vs 50%, p < 0.001). There was a non-significant trend towards identifying patients’ risk factors (58% vs 45%, p > 0.05) and it was noted that no unsafe prescriptions were recommended when risk factors were correctly identified.

Among eight general practitioners screening 79 patients, those randomly allocated to use a PDA (n = 4) with clinical guideline decision support demonstrated greater adherence to four out of five preventive healthcare guidelines, compared to colleagues who did not have access to a PDA. Although the small sample prevented statistical comparison, there was a pattern of greater improvement in rates of adherence among PDA users for including the prophylactic use of aspirin in patients at risk of coronary artery disease (from 33% before to 81% after PDA access), colorectal screening (38-65%) and screening for cervical cancer (88-100%) and cholesterol (64-94%). In comparison, there was a slight reduction in the high rate of screening for hypertension (97-94%) [16].

### Diagnostic decision making

There is evidence from three pilot RCTs [17-19] that having a clinical decision support system (CDSS) on a handheld computer can improve clinical and diagnostic decision making (Table 2). When used on a handheld computer in a clinical setting, the CDSS can prompt clinicians to collect and analyse patient data to inform diagnostic testing choices.

**Table 2:** Improved diagnostic decision making

**Diagnostic decision assessed**	**Percentage of episodes with PDA (n/n)**	**Percentage of episodes without PDA (n/n)**	**Percentage absolute difference (95% CI)**	**Number needed to test/** **screen**	**P Value**
Appropriate diagnostic strategy for pulmonary embolism [17]	55% 378/694	26% 245/951	29%	4	0.023
Adherence to recommended diagnostic testing [17]	41% 287/694	17% 162/951	24% (20-29)	5	0.030
Appropriate referral for cardiac stress testing at presentation [18]	49% 18/37	29% 8/28	20%	5	0.284
Appropriate referral for nuclear cardiology after cardiac stress testing [18]	63% 17/27	45% 5/11	18% (14-50)	6	0.4
Appropriate obesity-related diagnosis [19]	11% 91/807	1% 10/997	10% (8-13)	10	<0.05
Missed obesity-related diagnosis [19]	25% 51/208	67% 440/662	42% (35-48)	3	<0.05

For 24 emergency physicians, the use of a handheld CDSS led to greater improvement in diagnostic decision making for pulmonary emboli, compared to the use of paper guidelines [17]. While the use of a guideline improved appropriate diagnostic testing during the pre-intervention observational period, there was a greater clinical and statistically significant increase in appropriate diagnostic decision making by the physicians with a handheld CDSS during the intervention (p = 0.023). Physicians who used a CDSS on their PDA assessed a significantly greater proportion of patients’ pre-test probabilities and provided more appropriate diagnostic testing, especially for patients for whom pulmonary embolism was ruled out. When physicians in the handheld CDSS group recorded pre-test probabilities, they demonstrated greater adherence to recommended diagnostic testing (p = 0.03), and performed fewer mean tests per patient (p < 0.001). Therefore, in the assessment of pulmonary embolism in emergency departments, a handheld computer with a CDSS could provide a more appropriate diagnostic strategy for one out every four patients reviewed.

There was a non-significant trend towards more appropriate referral patterns to cardiac stress and nuclear cardiology testing, for patients with intermediate cardiac risk, among family physicians randomly assigned to use a PDA loaded with an interactive guideline for diagnosing angina, compared to customary care [18]. Further, those with the PDA and interactive guideline did not refer a higher percentage of patients on to cardiologists. It seems that use of the interactive guideline on a PDA could benefit one in every five patients referred for cardiac stress testing and one in six patients requiring additional nuclear cardiology testing.

When 13 advanced practice nurses were randomly allocated to use a CDSS that calculated an obesity-related diagnosis on their PDA, they identified significantly more obesity-related diagnoses (p < 0.05) and missed less obesity-related diagnoses (p < 0.05) than the control group who had a PDA but not the CDSS [19]. Further, one in ten patients with obesity could be more appropriately identified with obesity-related diagnoses and one in three patients may be prevented from having a missed diagnosis by providing nurses with a PDA and CDSS.

## Discussion

### Key findings

This systematic review provides a summary of current research evidence regarding the use of handheld computers to support clinical decision-making, from seven randomised controlled trials. We identified evidence suggesting that clinicians can effectively use handheld computers to access information to enhance their clinical knowledge, adhere to guidelines and make accurate and appropriate diagnostic decisions. The potential impact of clinicians using handheld computers to facilitate clinical decision making is high. Across six different diagnostic decisions, the numbers need to test/screen were all less than 11. If these results can be reproduced in larger studies, the practice benefits could be substantial.

It appears that a wide range of healthcare clinicians are engaging with multiple uses of handheld computers, in a complementary role to existing informational tools. Handheld computers can make synthesised information more accessible through the provision of detailed recommendations and across a range of conditions and settings. While one of the included studies demonstrated the benefit of providing GPs with patient-specific reminders about preventative guidelines at the point of care [16], a cluster randomised trial demonstrated that simpler strategies, such as text message reminders for outpatient paediatric malaria management, was associated with improved adherence to national guidelines in Kenya [20].

Quick access to accurate information is important in situations where the delivery of care becomes more complex [14]. It is likely that mobile CDSSs will enhance benefits already identified for computerised CDSSs, particularly for improving workflow efficiencies [21]. For example, when handheld computers are used with CDSSs, individual patient information can be integrated with synthesised research evidence to facilitate decision making at the point of care [22].

CDSSs are designed using either rule-based systems that represent knowledge in IF…THEN rules, or machine learning models where mathematical functions estimate risks given patient observations [10]. Both systems were referred to in the included studies. Clinicians were prompted to ask patients for specific contextual information most likely to impact their clinical diagnosis and treatment. This facilitated critical thinking around screening and management of patients [19]. Further, decision making was improved when excluding a diagnosis through more consistent documentation of pre-test probabilities and more appropriate use of tests for diagnostic investigations [17]. This review supports the mounting evidence from observational studies, that mobile CDSSs improve adherence to guidelines and policy, facilitate patient monitoring, provide valuable predictive tools, distinguish different levels of patient impairment and model medical problems for individualised care. Further, if they are integrated with electronic medical records, then individual patient data can be automatically included [10].

The evidence for using mobile CDSSs to directly improve patient outcomes remains sparse [3]. While the included studies do not provide statistically significant support for improved prescribing behaviour [13], there is convincing evidence from several large non-randomised studies. An observational study conducted within a rural US community randomised trial, demonstrated that a PDA-based CDSS steadily improved outpatient antibiotic prescribing rates within usual consultations [23]. Similarly, in a before/after prospective cohort trial in an Australian university-affiliated hospital’s intensive care unit (ICU), the use of a CDSS was associated with a reduction in antibiotic usage and patterns of use more consistent with clinical guidelines [24]. This study also demonstrated a decrease in mean patient length of stay in the ICU, which can be interpreted as a surrogate for patient outcomes and overall costs.

### Strengths and limitations

While this review focussed exclusively on RCTs, the conclusions are similar to the current and broader review of this topic [3]. Of the seven included studies, only four reported convincing, statistically significant evidence. This may represent the early proliferation of small and lower quality feasibility projects, associated with the growth of handheld computer use in healthcare [20]. This pattern is consistent with a recent broader review of mHealth technologies, in which none of the 42 included controlled trials were of high quality [3]. The heterogeneity of study designs and purposes makes the synthesis of this literature difficult. It will be important, in future studies, to be specific about the components of each intervention, so that the mechanisms of action and the impact of each component can be explored. However, the most promising results in both reviews were reported in the use of handheld computers for clinical management, appropriate testing and diagnosis.

As we did not search for studies via clinical trials registers, in other forms of grey literature, or published in other languages, there is a possibility that we may have missed unpublished studies. It is highly likely that participants in included studies were early adopters, who were more enthusiastic about the use of novel technology. Furthermore, there is reason to be concerned about publication bias given the sparse reporting of negative findings.

Although we did look to include smartphones and tablets in this review, we only found suitable trials of PDAs. It is also interesting to note that despite the proliferation of research into mobile technologies in health care, there were no RCTs published in the last 3 years sufficiently rigorous for inclusion in this analysis. However, it is expected that the functional benefits of smartphones and small tablets are likely to be consistent. As technology becomes more sophisticated and the range of software and apps increase, the way in which healthcare professionals use these resources is likely to build on the functionalities examined in the studies reported here.

### Implications for practice

With widespread adoption of handheld computers by healthcare professionals, there is potential for improved access to information and improved clinical decision making at the point of care. Handheld computers provide a tool for synthesising, organising and accessing a wide range of research evidence for use with individual patient data. While this review has identified effective use of handheld computers across a broad range of clinical situations, there is a need to demonstrate direct improvements in patient outcomes.

Further, there is a need to understand the conditions in which handheld computers work best [25]. It may be useful to conceptualise them as complex interventions, informed by a theory of behaviour change and supporting existing practices [4]. Understanding facilitators and barriers for their continued adoption is important. Potential facilitators could include high levels of access and motivation to use handheld technology and minimal training costs, using online and tailored training programmes. It will be necessary to highlight additional benefits and challenges as different user populations are studied, particularly in low and middle income countries. Further, partnerships between government, private investors and researchers will be important in developing mobile computing technology and its implementation strategies [4].

### Further research

The pace of technological change is moving faster than the time it takes to design, implement and report on rigorous research. However, there is evidence of a rise in the number of registered clinical trials of mHealth interventions in the USA [26]. Robust and novel research designs are required to rapidly evaluate the effectiveness of healthcare professionals using handheld computers to improve their access to information and their clinical decision making at the point of care.

It will be important to document how handheld computers can be integrated into normal work practices, and to demonstrate improved clinical outcomes such as prescribing rates and lengths of stay. It will also be important to carefully ‘blind’ participants to particular functions or apps if all clinicians are using handheld computers within their daily work routines.

Areas of high impact decision making such as emergency departments and intensive care units should be targeted for early RCTs. Following on, it will be important to broaden investigations across healthcare professionals in different clinical and geographic contexts, and to critically evaluate implementation plans and cost- benefit comparisons.

With the burgeoning development of apps, it will be important to monitor their accuracy and reliability [10]. It will also be important to monitor rates of handheld computer use in usual clinical care, and to supplement this with qualitative investigations of patient and provider attitudes and expectations. Further evaluation is warranted to investigate nurses’ perceptions that using PDAs in front of patients seemed rude and inconvenient [15].

## Conclusions

This review provides evidence that healthcare professionals’ use of handheld computers can improve their clinical decision making through improved information seeking and adherence to clinical guidelines. Handheld computers show promise for real time access to and analysis of clinical information, across many medical and health specialities. Handheld computers can host a variety of CDSS tools, which enable individual patient information to be integrated with synthesised research evidence, and facilitate decision making at the point of care. For diagnostic decisions, the numbers need to test/screen were all less than 11. However, to quantify costs and benefits for patients and healthcare systems, replication of these early results in more robust studies is urgently needed.

## Abbreviations

PDA: Personal digital assistant; SMS: Short message service; RCT: Randomized controlled trial; CDSS: Clinical decision support system; NSAID: Non-steroidal anti-inflammatory drugs; ICU: Intensive Care Unit.

## Competing interests

The authors declare that they have no competing interests.

## Authors’ contributions

JT and SM conceived and designed the study; NR designed and implemented the search strategy; JT and SM screened titles and abstracts; HA and SM read full texts, assessed quality and extracted data from included studies, and prepared the manuscript. All authors contributed to editing the manuscript, read and approved the final manuscript.

## Pre-publication history

The pre-publication history for this paper can be accessed here:

http://www.biomedcentral.com/1472-6947/14/56/prepub

## Supplementary Material

Additional file 1 Medline search strategy. Click here for file
