# Supplementary material for: Use of handheld computers in clinical practice: a systematic review
Source: BMC Med Inform Decis Mak. 2014 Jul 6;14:56. doi: 10.1186/1472-6947-14-56 (PMC4099138; doi:10.1186/1472-6947-14-56)
Supplement: Additional file 1 — Medline search strategy. [file 1472-6947-14-56-S1.doc]

**Appendix one: Medline Search Strategy**

How are hand held computing devices useful for clinicians in supporting their clinical practice?

**Medline (OvidSP)**

1. exp Health Personnel/

2. exp health occupations/

3. clinician*1.ti,ab.

4. (clinical adj3 (staff or personnel or officer*1 or worker*1)).ti,ab.

5. (medical adj3 (staff or personnel or officer*1 or worker*1 or professional*)).ti,ab.

6. ((health or healthcare) adj3 (staff or personnel or officer* or worker*1 or professional*1)).ti,ab.

7. (physician*1 or doctor*1 or surgeon*1).ti,ab.

8. nurse*1.ti,ab.

9. practitioner*1.ti,ab.

10. (physical therapist*1 or physiotherapist*1).ti,ab.

11. rehabilitation therapist*1.ti,ab.

12. occupational therapist*1.ti,ab.

13. ((speech or language) adj3 therapist*).ti,ab.

14. 1 or 2 or 3 or 4 or 5 or 6 or 7 or 8 or 9 or 10 or 11 or 12 or 13

15. Computers, Handheld/

16. (mhealth* or m-health* or mobile health*).ti,ab.

17. (handheld adj3 (device* or computer* or technolog* or PC)).ti,ab.

18. (hand-held adj3 (device* or computer* or technolog* or PC)).ti,ab.

19. (tablet adj3 (device* or computer* or technolog* or PC)).ti,ab.

20. (slate adj3 tablet*).ti,ab.

21. (android adj3 tablet*).ti,ab.

22. (palm*1 adj3 (device* or computer* or pc)).ti,ab.

23. (pocket* adj3 (device* or computer* or pc)).ti,ab.

24. (mobile adj3 (computer* or PC)).ti,ab.

25. (portable adj3 (computer* or PC)).ti,ab.

26. (palm pilot* or palmpilot*).ti,ab.

27. (smartbook* or smart-book*).ti,ab.

28. ipad*.ti,ab.

29. i-pad*.ti,ab.

30. galaxy tab.ti,ab.

31. kindle fire.ti,ab.

32. playbook.ti,ab.

33. ((touchscreen or touch screen) adj3 (computer* or PC or technolog* or device*)).ti,ab.

34. (smartphone* or smart phone*).ti,ab.

35. iphone*.ti,ab.

36. i-phone*.ti,ab.

37. (blackberry or black-berry).ti,ab.

38. (android adj3 (phone* or cellphone* or telephone* or device* or mobile*)).ti,ab.

39. (google adj3 (phone* or cellphone* or telephone* or device* or mobile*)).ti,ab.

40. (nexus one adj3 (phone* or cellphone* or telephone* or device* or mobile*)).ti,ab.

41. (google adj3 android).ti,ab.

42. (ipod touch or i-pod touch).ti,ab.

43. ((touchscreen or touch screen) adj3 (phone* or cellphone* or telephone*)).ti,ab.

44. apps.ti.

45. or/15-44

46. Cellular Phone/

47. ((cell or cellular) adj3 (phone* or telephone*)).ti,ab.

48. cellphone*.ti,ab.

49. (mobile adj3 (phone* or telephone* or device* or technolog*)).ti,ab.

50. digital assistant*.ti,ab.

51. pda*.ti,ab.

52. 46 or 47 or 48 or 49 or 50 or 51

53. exp Internet/

54. Software/

55. Wireless Technology/

56. internet.ti,ab.

57. (online or on-line).ti,ab.

58. (web* or www).ti,ab.

59. (wireless or wifi or wi-fi or wap).ti,ab.

60. (bluetooth or blue tooth).ti,ab.

61. 53 or 54 or 55 or 56 or 57 or 58 or 59 or 60

62. 52 and 61

63. 45 or 62

64. 14 and 63
